# Robust Bayesian multilevel meta-analysis: Adjusting for publication bias in the presence of dependent effect sizes

**DOI:** 10.3758/s13428-026-03023-y

**Published:** 2026-05-12

**Authors:** František Bartoš, Maximilian Maier, Eric-Jan Wagenmakers

**Affiliations:** 1 Department of Psychological Methods, University of Amsterdam, Amsterdam, Netherlands; 2 Institute of Economic Studies, Faculty of Social Sciences, Charles University, Prague, Czechia; 3 Department of Experimental Psychology, University College London, London, England; 4 Behavioural Sciences Group, Warwick Business School, University of Warwick, Coventry, England

**Keywords:** Bayes factor, Meta-analysis, Knowledge updating, Evidence, Bayesian model averaging

## Abstract

Meta-analyses often include multiple dependent effect sizes, yet current methods typically neglect the resulting within-study dependencies or fail to address model uncertainty and publication bias adequately. We extend robust Bayesian meta-analysis (RoBMA) to a multilevel framework, simultaneously handling within-study dependencies, model uncertainty, heterogeneity, moderators, and publication bias. Specifically, the three-level RoBMA integrates approximate Bayesian selection models with PET-PEESE adjustments within a hierarchical Bayesian setting. We illustrate the methodology through empirical examples and demonstrate its performance via simulations. The approach is implemented in the RoBMA R package and JASP.

Meta-analysis and meta-regression allow researchers to combine effect sizes across multiple studies and explore between-study heterogeneity. However, individual studies often provide multiple, dependent effect sizes, violating the independence assumption central to fixed-effect and random-effects meta-analysis (Borenstein, Hedges, Higgins, & Rothstein, 2009). Such violations yield overly narrow confidence intervals and inflated statistical evidence, which manifests as elevated false-positive rates in frequentist tests or inflated Bayes factors. Indeed, a recent review of 1000 meta-analyses across ten disciplines found that 57% included multiple effect sizes per study, highlighting the widespread nature of this issue (Wu, Duan, Reed, & Tipton, 2025, also see Nakagawa, Yang, Macartney, Spake, & Lagisz, 2023; Tipton, Pustejovsky, & Ahmadi, 2019; Yang, Macleod, Pan, Lagisz, & Nakagawa, 2023).

Violations of the independence assumption typically fall into two categories, each requiring a distinct methodological response. Firstly, studies may collect multiple independent samples (often participants), where shared procedures, participant populations, or experimental designs induce within-study dependencies not fully captured by moderators. For example, a researcher may report effect size estimates of the relationship between motivation and performance from three independent experiments. Such dependencies are usually addressed via multilevel or three-level meta-analysis (e.g., Konstantopoulos, 2011; Van den Noortgate, López-López, Marın-Martınez, & Sánchez-Meca, 2013).^1^ Secondly, studies may use a single sample but report multiple outcomes or comparisons involving a common control group, resulting in statistical dependencies among effect sizes. For example, a researcher may report effect size estimates of the relationship between motivation and performance using three different performance scales measured on the same sample of participants. These cases are typically addressed using multivariate meta-analysis (e.g., Olkin & Gleser, 2009) or by averaging effect size estimates (e.g., Borenstein et al., 2009; Tipton, 2015).^2^ In this manuscript, we focus on the first type of dependency—multiple effect sizes nested within studies—and refer to it as ‘within-study dependency.’

Existing meta-analysis approaches aiming to account for within-study dependency come with several limitations. First, they usually rely on frequentist testing and *p*-values and thus cannot distinguish between ‘absence of evidence’ (i.e., when the data are not diagnostic) and ‘evidence of absence’ (i.e., when the data provide support for the null-hypothesis; e.g., Wagenmakers, 2007 and Keysers, Gazzola, & Wagenmakers, 2020). Second, researchers need to base the inference on a single model (with the exception of pseudo Bayesian model-averaging techniques, Burnham & Anderson, 2002; Steel, 2020; Harrer, Cuijpers, A, & Ebert, 2021) and thus fail to incorporate the uncertainty inherent in the initial model selection step (Wagenmakers, Sarafoglou, & Aczel, 2022). Finally, adequate adjustment for publication bias is crucial as unadjusted estimates tend to overestimate the evidence for and size of effects (e.g., Kvarven, Strømland, & Johannesson, 2020; Bartoš et al., 2024; Bartoš et al., 2023; Fanelli, Costas, & Ioannidis, 2017; Fanelli, 2010; Stanley, Carter, & Doucouliagos, 2018; Ioannidis, Stanley, & Doucouliagos, 2017; van Aert, Wicherts, & Van Assen, 2019; Schwab, Kreiliger, & Held, 2021). In multilevel settings, adjustments for publication bias (i.e., selection at the study-level) and adjustments for selective outcome reporting (i.e., selection at the estimate level) have so far been studied and accomplished only by regression-based approaches (Chen & Pustejovsky, 2025, but see a novel selection model approach proposed by Pustejovsky, Citkowicz, & Joshi 2025, and tests for the presence of publication bias by Rodgers & Pustejovsky, 2021; Fernández-Castilla et al., 2021; Park, Beretvas, & Smith, 2025); however, these approaches have drawbacks in certain meta-analytic situations (Lau, Ioannidis, Terrin, Schmid, & Olkin, 2006). It is therefore important to extend meta-analysis models to account for other ways in which publication bias may operate, such as selection based on *p* value thresholds (i.e., selection models; Vevea & Hedges, 1995).

We address these limitations by extending robust Bayesian meta-analysis (RoBMA; e.g., Maier, Bartoš, & Wagenmakers, 2023; Bartoš, Maier, Wagenmakers, Doucouliagos, & Stanley, 2022b) to multilevel settings. RoBMA uses Bayesian model averaging to incorporate uncertainty about the underlying data-generating process into meta-analytic inference. Specifically, RoBMA integrates different model assumptions regarding the presence of an effect, heterogeneity, and publication bias (e.g., Hinne, Gronau, van den Bergh, & Wagenmakers, 2020; Fragoso, Bertoli, & Louzada, 2018; Hoeting, Madigan, Raftery, & Volinsky, 1999). Importantly, RoBMA incorporates multiple methods for publication bias adjustment, including PET-PEESE (a model that assumes a linear and quadratic relationship between effect sizes and standard errors; e.g., Stanley & Doucouliagos, 2014) and selection models (assuming selection based on *p* value thresholds; e.g., Vevea & Hedges, 1995). Through Bayesian model-averaging, RoBMA evaluates a comprehensive ensemble of models, weighting each according to its predictive performance as measured by the marginal likelihood (the probability of the data given the model). Thus, RoBMA allows the data itself to determine the appropriate degree of reliance on each model, making the approach more robust to misspecification. Previous studies demonstrated RoBMA’s superior performance in adjusting for publication bias, as evidenced by both simulation and empirical evaluations (Maier et al., 2023; Bartoš et al., 2022b). Further, we combine the three-level extension of RoBMA with a recent extension to meta-regression analysis (Bartoš, Maier, Stanley, & Wagenmakers, 2025a), allowing for meta-regression analysis when effects are nested within primary studies. By extending RoBMA and RoBMA meta-regression to accommodate within-study dependencies, researchers confronted with this complication can now take advantage of RoBMA’s ability to quantify Bayes factor evidence (Jeffreys, 1935, 1939; Kass & Raftery, 1995) both for and against the presence of effects, heterogeneity, moderation, and publication bias; moreover, they can systematically account for model uncertainty via Bayesian model averaging (Hinne et al., 2020; Hoeting et al., 1999). These advantages come at a cost. The methodology is more complex and requires more computational resources than the frequentist counterparts (in orders of tens of minutes to hours). Unlike for frequentist methods, analysts further have to specify prior parameter distributions and prior model probabilities to fully define the models.

In the remainder of this manuscript, we first outline a multilevel framework for robust Bayesian meta-analysis. Second, we illustrate the methodology on two examples: a meta-analysis by Johnides, Borduin, Sheerin, and Kuppens (2025) on secondary benefits of family member participation in treatments for childhood disorders and a meta-regression example by Havránková, Havránek, Bortnikova, and Bartoš (2025) on the relationship between perceived beauty and professional success. Alongside these sections, we also refer to additional literature that provides the necessary background to navigate the Bayesian methodology. Finally, we demonstrate the performance of multilevel robust Bayesian meta-analysis in two simulation studies. First, we extend a recently published simulation study (Chen & Pustejovsky, 2025) to evaluate the method’s performance in three-level settings. Second, we designed a narrower simulation study to evaluate meta-regression analysis within three-level settings. We also implemented the multilevel robust Bayesian meta-analysis and meta-regression (as well as the simpler, publication bias unadjusted versions of those models) in the RoBMA R package (Bartoš & Maier, 2020) and the free and open-source statistical software JASP (JASP Team, 2025). These software implementations make it relatively easy for researchers to draw robust Bayesian meta-analytic inferences while accounting for within-study dependencies.

## Method

### Robust Bayesian meta-analysis

Robust Bayesian meta-analysis builds upon a conventional random-effects model, assuming both the existence of a common meta-analytic effect $\mu$ and between-study heterogeneity $\tau$. Formally, given *K* observed effect sizes $\text {y}_i$ with standard errors $\text {se}_i$, the model is specified as

1 $$\begin{aligned} \text {y}_i \sim \text {Normal}(\mu , \text {se}_i^2 + \tau ^2). \end{aligned}$$

This standard random-effects model can be extended into a meta-regression by allowing each study-specific effect size parameter $\theta _i$ to depend on study characteristics $x_{i,k}$, with corresponding regression coefficients $\beta _k$ (Baker et al., 2009; Stanley & Jarrell, 1989),

2 $$\begin{aligned} \nonumber \theta _i&= \mu + \beta _1 x_{i1} + \dots + \beta _K x_{iK},\\ \text {y}_i&\sim \text {Normal}(\theta _i, \text {se}_i^2 + \tau ^2). \end{aligned}$$

See Bartoš et al. (2025a) for a detailed treatment of the Bayesian meta-regression framework as implemented in RoBMA. Apart from including moderators, meta-regression allows for PET- or PEESE-type adjustments for publication bias, by modeling linear (PET) or quadratic (PEESE) relationships between effect sizes and their standard errors, incorporating $\text {se}_i$ and $\text {se}_i^2$ directly as predictors (Stanley & Doucouliagos, 2014; Bartoš et al., 2022b).

The basic model from Eq. 1 can also incorporate step-function selection model adjustments by replacing the normal likelihood with a weighted likelihood that includes relative publication probabilities $\omega$ (e.g., Silliman, 1997; Larose & Dey, 1998; Guan & Vandekerckhove, 2016):

3 $$\begin{aligned} \text {y}_i \sim \text {Weighted-Normal}(\mu , \text {se}_i^2 + \tau ^2, \omega ). \end{aligned}$$

Moreover, selection model adjustments can be combined with meta-regression (Eq. 2) to yield ‘selection model meta-regression’ (Bartoš et al., 2025a). Lastly, by constraining relevant parameters to zero, nested models can represent scenarios where effects, heterogeneity, or moderation are absent.

In earlier work, we described how RoBMA systematically combines models representing different assumptions about the presence of a treatment effect, heterogeneity, moderation, and publication bias through Bayesian model averaging (Maier et al., 2023; Bartoš et al., 2022b; Bartoš et al., 2025a; Fragoso et al., 2018; Raftery, Madigan, & Volinsky, 1995; Hinne et al., 2020). This approach yields model-averaged parameter estimates and inclusion Bayes factors, explicitly quantifying evidence for or against specific model components. However, these previous formulations of RoBMA did not account for multilevel data structures (i.e., multiple dependent effect sizes within studies). We generalize RoBMA to multilevel settings in the following subsection.

#### Multilevel robust Bayesian meta-analysis

In order to extend RoBMA to multilevel settings, we build upon the multilevel meta-analytic framework (extended to meta-regression) as outlined in Konstantopoulos (2011). Specifically, the observed effect sizes are assumed to be multivariate normally distributed with mean vector $\boldsymbol{\theta }$ and block-diagonal covariance matrix $\Sigma$ (composed of between-study heterogeneity $\tau _\gamma$ and within-study heterogeneity $\tau _\psi$), and the sampling diagonal variance-covariance matrix $\text {S}^2$ (composed of the effect size standard errors $\text {se}_{ij}$). The block-diagonal covariance matrix allows us to separate the full-sample multivariate normal distribution into $i = 1, \dots , I$ study-specific independent multivariate normal distributions, with mean sub-vectors $\boldsymbol{\theta _i}$ and variance-covariance sub-matrices $\Sigma _i + \text {S}_i$ for $j = 1, \dots , J_i$ effect size estimates.

4 $$\begin{aligned} \text {y}_{i}&\sim \text {Multivariate-Normal}(\boldsymbol{\theta }_i, \text {S}_i + \Sigma _i)\\ \nonumber \text {y}_{i}&= [\text {y}_{i1}, \text {y}_{i2}, \dots , \text {y}_{iJ_i} ] \\ \nonumber \boldsymbol{\theta _i}&= [\theta _{i1}, \theta _{i2}, \dots , \theta _{iJ_i} ] \\ \nonumber \text {S}_{i} + \Sigma _i&= \begin{bmatrix} \tau _\psi ^2 + \tau _\gamma ^2 + \text {se}_{i1}^2 & \tau _\psi ^2 & \dots & \tau _\psi ^2 \\ \tau _\psi ^2 & \tau _\psi ^2 + \tau _\gamma ^2 + \text {se}_{i2}^2 & \dots & \tau _\psi ^2 \\ \dots & \dots & \dots & \dots \\ \tau _\psi ^2 & \tau _\psi ^2 & \dots & \tau _\psi ^2 + \tau _\gamma ^2 + \text {se}_{iJ_i}^2 & \\ \end{bmatrix}. \end{aligned}$$

While the marginalized parameterization in Eq. 4 works well for multilevel meta-regression and PET-PEESE adjustments, it becomes prohibitively complex for multilevel selection models where the multivariate normal distribution is replaced by a weighted-multivariate normal distribution.^3^

To overcome these computational hurdles, we adopt a non-marginalized Bayesian hierarchical parameterization commonly used in Bayesian statistics (e.g., Gelman & Hill, 2006; Lee & Wagenmakers, 2005; McElreath, 2020). Under this approach, between-study heterogeneity $\tau _\gamma$ is explicitly represented via study-level random effects $\gamma _i$, thereby decomposing the multivariate normal structure from Eq. 4 into independent normal distributions,

5 $$\begin{aligned} \gamma _i&\sim \text {Normal}(0, \tau _\gamma ^2) \\ \nonumber \text {y}_{ij}&\sim \text {Normal}(\theta _{ij} + \gamma _i, \text {se}_{ij}^2 + \tau _\psi ^2). \end{aligned}$$

We retain the marginalization of within-study heterogeneity $\tau _\psi$ so as to limit the impact of random effects misspecification. Importantly, Eqs. 4 and 5 are equivalent only if the normal distribution for the observed effect sizes is assumed. In the case of selection models, the extension of Eq. 4 into weighted-multivariate-normal parameterization would allow for propagating the selection effects through the within and between study heterogeneities—the distribution of $\gamma _i$ is not normal as some of the study-specific random effects would be omitted due to the publication bias. The extension of Eq. 5 into weighted-normal parameterization

6 $$\begin{aligned} \gamma _i&\sim \text {Normal}(0, \tau _\gamma ^2) \\ \nonumber \text {y}_{ij}&\sim \text {Weighted-Normal}(\theta _{ij} + \gamma _i, \text {se}_{ij}^2 + \tau _\psi ^2, \omega ). \end{aligned}$$

does not allow the propagation of the selection effects to the between-study heterogeneity (i.e., the model assumes that the selection happens on the estimate-level rather than the study-level). Consequently, the distribution for the between-study random effects $\gamma _i$ is misspecified if both studies (publication bias) and effects (selective outcome reporting) are selected. Notably, despite this misspecification, the subsequent simulation study demonstrates that our multilevel formulation outperforms standard (single-level) robust Bayesian meta-analysis in contexts involving dependent effect sizes.

The development of the Bayesian multilevel meta-regression-based adjustments (for the PET and PEESE model) and the approximate selection models allows us to retain the same set of publication bias adjustment components

1. Two-sided selection models
  - *p* value cutoffs = 0.05
  - *p* value cutoffs = 0.05 & 0.10
2. One-sided selection models
  - *p* value cutoffs = 0.05
  - *p* value cutoffs = 0.025 & 0.05
  - *p* value cutoffs = 0.05 & 0.50
  - *p* value cutoffs = 0.025 & 0.05 & 0.50
3. Models adjusting for the relationship between effect sizes and standard errors
  - PET model
  - PEESE model

as was used in the standard RoBMA model ensemble (see Table 1 in Bartoš et al., 2022b for a complete description of the RoBMA model ensemble, including information about prior distributions).

### Prior distributions

The multilevel extension of RoBMA replaces the (overall) heterogeneity parameter $\tau$ with two parameters representing within-study heterogeneity ($\tau _\psi$) and between-study heterogeneity ($\tau _\gamma$). Default priors and guidance on specifying custom priors for effect sizes, publication bias parameters, and moderators remain consistent with previous RoBMA implementations (for detailed recommendations see Maier et al., 2023; Bartoš et al., 2022b; Bartoš, Maier, Quintana, & Wagenmakers, 2022a; Bartoš et al., 2025a). More general guidance on specifying prior distributions suitable for Bayesian hypothesis testing and model-averaging is also available in Mulder and van Aert (2025) and Bartoš and Wagenmakers (2025) and references therein.

We specify prior distributions for the within-study ($\tau _\psi$) and between-study ($\tau _\gamma$) heterogeneity by decomposing the total heterogeneity parameter $\tau$ from standard RoBMA into a within/between allocation via a heterogeneity allocation parameter $\rho$,

7 $$\begin{aligned} \tau _\psi ^2&= \rho \tau ^2 \\ \nonumber \tau _\gamma ^2&= (1-\rho ) \tau ^2, \end{aligned}$$

mirroring the parameterization in Konstantopoulos (2011). In contrast to classical meta-analytic approaches that first require heterogeneity estimates before computing the remaining meta-analytic parameters (Hedges & Olkin, 2014), the Bayesian approach with Markov chain Monte Carlo (MCMC) sampling allows us to estimate all parameters jointly (in one go) and propagate their uncertainty through the model.

The only additional prior distribution required for three-level RoBMA is the prior on the heterogeneity allocation parameter $\rho$. Given that researchers typically do not have strong prior expectations regarding this allocation, and because our primary focus is not explicitly on testing the existence of nested structures, we recommend a uniform Beta(1,1) prior on the [0,1] interval as a sensible default choice. This default can be easily adjusted within the RoBMA R package, such that users may incorporate more informative prior distributions and explicitly test for the presence versus absence of within-study heterogeneity (where $\rho =0$ corresponds to within-study independence). For details and guidance, we refer readers to the vignette section “Multilevel meta-analysis” https://cran.r-project.org/web/packages/RoBMA/vignettes/v10-metafor-parity-multilevel.html.

## Examples

We present two examples in this section. The first example concerns a re-analysis of a meta-analysis on secondary benefits of family participation in treatment for childhood disorders. This example demonstrates how to apply and interpret multilevel RoBMA in R and JASP. The second example concerns a re-analysis of a meta-regression of the relationship between beauty and professional success. This example demonstrates how to apply and interpret multilevel RoBMA meta-regression in R. In addition, while the first example uses standardized mean differences, which allows us to directly apply default prior distributions as developed in the previous manuscripts, the second example relies on a non-standardized effect size, which requires us to rescale the default prior distributions. For brevity, we fully report only the multilevel RoBMA meta-analysis and meta-regression results and briefly compare their results to other publication bias-unadjusted and -adjusted methods later introduced in the simulation study (Tables 1 and 2 in the Appendix). Bayes factors are interpreted according to the standard conventions (1 < BF < 3 indicates weak evidence, 3 < BF < 10 indicates moderate evidence, and BF > 10 indicates strong evidence, see Jeffreys, 1939; Lee & Wagenmakers, 2013). Reproducible R vignettes and annotated JASP analysis files are available at https://osf.io/nd2gv/.

### Secondary benefits of family participation in treatment for childhood disorders

Johnides et al. (2025) conducted a meta-analysis to investigate the extent to which family-based treatments for children with mental health, physical health, and developmental disorders provide secondary benefits to the children’s siblings and caregivers. Using a three-level meta-analysis examining 412 effect sizes from 128 studies, Johnides et al. (2025) found an average effect size of family-based treatments on secondary benefits of $d = 0.25$, 95% CI [0.19, 0.30] (relative to individually focused treatments). Subsequent publication bias adjustment analyses failed to find publication bias using a standard three-parameter selection model (3PSM) $\chi ^2(1) = 1.87$, $p = 0.17$. However, moderation analyses revealed a statistically significant relationship with sample size—suggestive of potential publication bias due to systematic associations between effect sizes and standard errors, as discussed by Johnides et al. (2025).

**Fig. 1:**
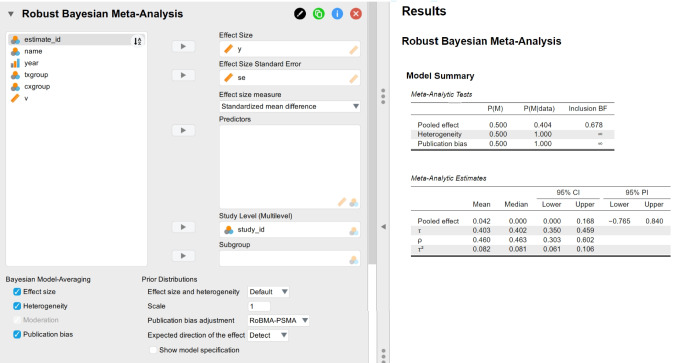
Results from Johnides et al. (2025) using the multilevel robust Bayesian meta-analysis in JASP

We revisit this analysis using the newly developed multilevel RoBMA, improving upon the original analysis in two key aspects. First, RoBMA comprehensively adjusts for multiple forms of publication bias, including selection on marginally significant *p* values and selection for positive effects (i.e., suppression of negative or “backfire” effects). It combines results from different publication bias-adjustment methods proportionally to their prior predictive performance. As such, the inference is more heavily based on models that predicted the data well, while models that predicted the data poorly are discounted. Second, unlike the previously employed 3PSM, multilevel RoBMA explicitly accounts for the nested three-level structure of the data while simultaneously addressing publication bias. The code snippet below illustrates how to estimate the model in R using the RoBMA package (version 3.6.1, Bartoš & Maier, 2020),

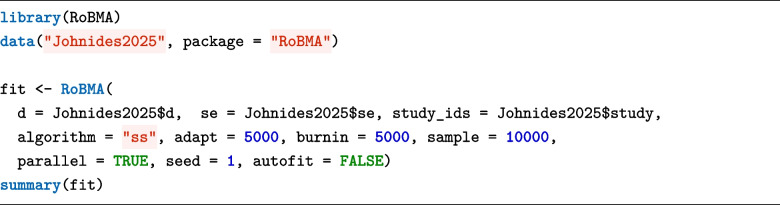



and Fig. 1 visualizes the graphical user interface and the results of the multilevel robust Bayesian meta-analysis when performed via the Meta-Analysis module in JASP. To conduct the analysis, users can specify the corresponding “Effect Size”, “Effect Size Standard Error”, and “Study Level (Multilevel)” variables from the dataset in the left input panel. The right output panel of Fig. 1 summarizes the inclusion Bayes factors for the presence of the effect, heterogeneity, and publication bias as well as the pooled meta-analytic estimates. (JASP Team, 2025). The meta-analysis model usually takes about 5–15 min to run with parallel processing enabled in R, depending on CPU performance, but the current version of JASP relies on single-threaded processing and usually takes about four times as long to estimate the model.

Application of the multilevel RoBMA to the data from Johnides et al. (2025) reveals weak evidence against the presence of the effect ($\text {BF}_{10} = 0.927$) and extreme evidence for the presence of both between-study heterogeneity and publication bias (both $\text {BFs} > 10^6$). The model-averaged posterior mean effect estimate of $d = 0.050$, 95% CI [0.000, 0.173] is accompanied by substantial overall heterogeneity $\tau = 0.403$, 95% CI [0.350, 0.460]. This very large heterogeneity estimate is reflected in a wide 95% prediction interval ranging from $-0.752$ to 0.845. As such, despite the pooled effect estimate being close to zero, we cannot rule out the possibility of many true effects being both positive and negative. Finally, the estimated heterogeneity allocation parameter $\rho = 0.461$, 95% CI [0.306, 0.603] suggests slightly greater within-study heterogeneity compared to between-study heterogeneity.

These findings contrast markedly with the original unadjusted effect size estimate and the non-significant publication bias test (3PSM). Furthermore, RoBMA reconciles the conflicting results from the publication bias-unadjusted methods, selection model, and regression-based approaches (Table 1 in the Appendix). While random-effects models, 3PSM, and 4PSM reject the null hypothesis of no effect and suggest positive treatment effects, the regression-based approaches adjust the effect estimate to zero. In contrast to the single-level RoBMA, which would find strong evidence for the absence of the effect, accounting for the nested nature of the data provides a more conservative assessment and only weak evidence for the absence of the effect.

**Fig. 2:**
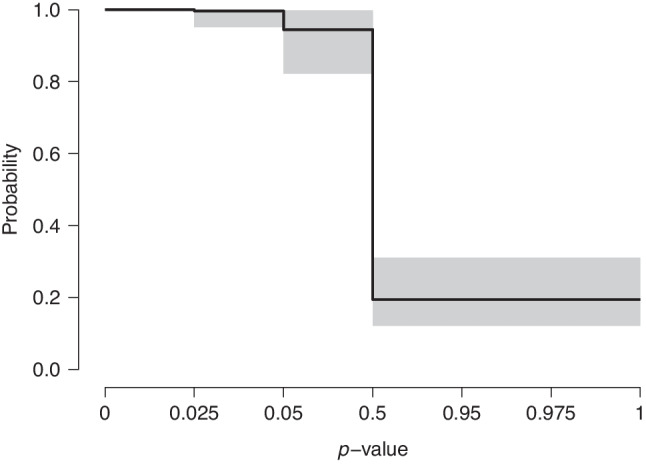
Multilevel robust Bayesian meta-analysis reveals severe selection for positive effects in the meta-analysis of Johnides et al. (2025). *Note.* The *x*-axis is rescaled to equal spacing between the one-sided *p* values thresholds

Further inspection of the RoBMA results clarifies that the identified publication bias does not arise from small-study effects (i.e., the relationship between effect sizes and their standard errors); instead, the more flexible weight functions incorporated in RoBMA obtain all posterior model probability: the selection specifically favors positive effects and there is a substantially lower probability of publishing negative results (relative probability = 0.198, 95% CI [0.122, 0.317]; see Fig. 2 for the weight function).

### Relationship between beauty and professional success

Havránková et al. (2025) conducted a meta-analysis and meta-regression of field studies investigating the relationship between perceived beauty and professional success. The effect was quantified as the percent increase in earnings per standard deviation increase in beauty. In this example, we re-examine a meta-regression assessing whether this relationship depends on the type of contact with the consumer that the job required, characterized as either “no customer contact”, “some customer contact”, or “direct customer contact”. The original meta-regression was performed on 1159 effect sizes from 67 studies and employed a wide range of publication-bias adjustment methods (including multilevel RoBMA).

To apply the Bayesian methodology, we need to rescale our default prior distributions. This is necessary since the effect sizes are not standardized (i.e., they quantify the relationship as the percent increase in earnings with a one-standard-deviation increase in beauty; instead of a standardized effect size such as a standardized mean difference (Cohen’s *d*/Hedges’ *g*), log odds ratio, or correlation). If we were to use the default prior distributions without any modification, we would risk specifying overly narrow prior distributions (which would make it difficult to find evidence for either the presence or absence of the effect since all models would predict very small effect sizes) or overly wide prior distributions (which would be biased towards the null hypothesis since the alternative models would predict unrealistically large effects; see Wagenmakers & Ly, 2023).

**Fig. 3:**
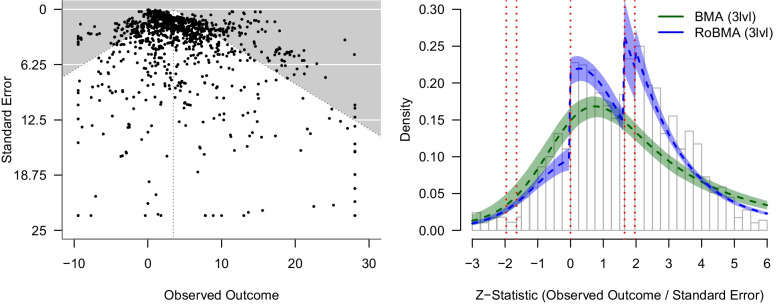
Funnel plot and meta-analytic *Z*-curve plot indicating publication bias in a meta-analysis of the relationship between beauty and professional success. *Note.* Data from Havránková et al. (2025). The meta-analytic *Z*-curve plot compares the model fit of a multilevel Bayesian model-averaged meta-regression (*green*) and multilevel robust Bayesian model-averaged meta-regression (*blue*). Five and 44 test statistics are below and above the plotting range, respectively

The rescaling can be easily achieved by matching the scale of the default prior distributions specified in Bartoš et al. (2022b) to the unit information scale of the data (e.g., Röver et al., 2021; Mulder & van Aert, 2025). The unit information scale of the data can be obtained by taking a product of the standard error of a fixed-effect meta-analytic model and the square root of the total sample size (see Eq. 6 in Röver et al., 2021). The default prior distribution for the effect size specified on standardized mean differences has a standard deviation of one; the default prior scale therefore corresponds to one-half of the unit information (Chapter 2.4, Spiegelhalter, Abrams, & Myles, 2004). As such, the unit information scale of the data must be multiplied by one-half, as shown in the code snippet below.

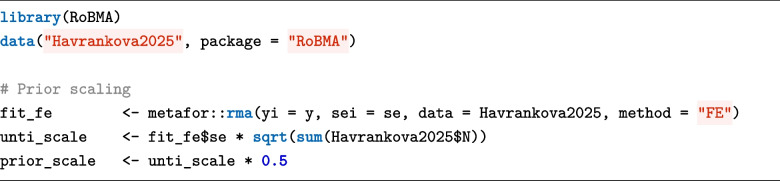



The resulting prior_scale, in our example $\approx 26$, can be used in the rescale_priors argument in the fitting functions of the RoBMA package,

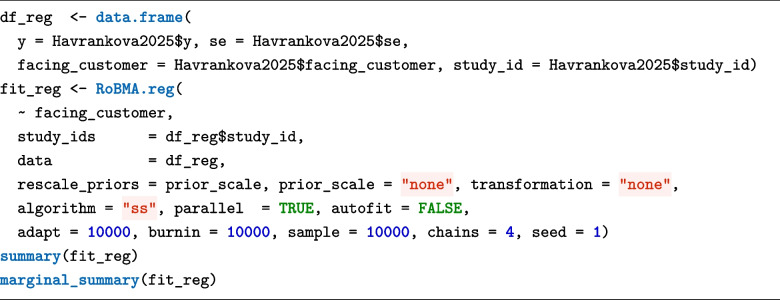



where we first specify the input data.frame (with y and se denoting the effect size and standard error inputs) and prior_scale = "none" and transformation = "none" arguments disable prior distributions for standardized effect sizes. The summary and marginal_summary functions provide the overall model estimates and the estimated marginal means. The meta-regression model can take 1-2 hours to run with parallel processing enabled ($4\times$ as long in JASP without parallel processing). Also see Bartoš and Wagenmakers (2025) for more details on (publication bias) adjusted Bayesian (model-averaged) meta-regression models in JASP.

Using the rescaled prior distributions, the multilevel RoBMA meta-regression finds extreme evidence for the presence of the average effect, moderation by the degree of consumer contact, between-study heterogeneity, and publication bias (all $\text {BFs} > 10^6$). The model-averaged average effect estimate of $\mu = 3.0$, 95% CI [1.9, 4.2], is accompanied by considerable overall heterogeneity $\tau = 4.5$, 95% CI [3.9, 5.4], and within-cluster allocation close to 1, $\rho = 0.82$, 95% CI [0.75, 0.88], indicating a high degree of similarity of estimates from the same study. Importantly, the average effect is moderated by the degree of consumer contact; examining the estimated marginal means at each level of the moderator reveals moderate evidence for the absence of the effect for no consumer contact jobs $\mu = 0.8$, 95% CI $[-0.6, 2.2]$, $\text {BF}_{10} = 0.105$, and extreme evidence for jobs with some consumer contact $\mu = 4.0$, 95% CI [2.5, 5.6], $\text {BF}_{10} > 10^6$ and jobs with direct consumer contact $\mu = 4.2$, 95% CI [3.0, 5.6], $\text {BF}_{10} > 10^6$.

Comparison to other publication bias-unadjusted and -unadjusted methods (Table 2 in the Appendix) again reveals considerable divergence in the results. For example, apart from 3PSM, the remaining publication bias-adjusted models tend to shrink the average effect close to zero and often find evidence for the null hypothesis (single-level RoBMA) or fail to reject the null hypothesis (multilevel PET-PEESE/EK). From the publication bias-unadjusted models, all but the random-effects meta-analysis with correlated hierarchical effects with inverse sampling covariance weights (RE CHE-ISCW), find approximately similar average effects. With respect to the test of moderation, only selection models reject the null hypothesis of no moderation and multilevel BMA meta-regression and single-level RoBMA find evidence for the presence of moderation. Random-effects models and regression-based methods fail to reject the null hypothesis. Only with respect to publication bias, the different methods tend to agree on rejecting the null hypothesis of the absence of publication bias/finding extreme evidence for the presence of publication bias.

To resolve some of these differences, we first turn to Fig. 3. The left panel visualizes the classical funnel plot (left), which is somewhat indicative of a right skew (with a statistically significant Egger’s test, $p < 0.001$); that is also detected by the multilevel regression-based methods (see Table 2). The right panel visualizes the meta-analytic z-curve plot (Bartoš & Schimmack, 2025). The z-curve plot shows the distribution of the observed *z*-statistics (computed as the effect size / standard error), with dotted red horizontal lines highlighting the typical steps on which publication bias operates ($z = \pm 1.65$ and $z = \pm 1.96$ corresponding to statistically significant and marginally significant *p*-values, and $z = 0$ corresponding to the selection of the direction of the effect). We can notice clear discontinuities corresponding to the selection on the direction of the effect and marginal significance. The posterior predictive densities from multilevel BMA meta-regression (green) and multilevel RoBMA meta-regression (blue) clearly indicate that while RoBMA approximates the observed data reasonably well, the publication bias unadjusted models miss those discontinuities and notably underestimate the proportion of just statistically significant results (while overestimating the proportion of studies with very high *z*-statistics, outside of the plotting range).

Second, a further exploration of the data revealed that studies with smaller average effects contribute more estimates than studies with larger average effects, with a correlation between the log cluster size and the average cluster effect of $r = -0.23$. We suspect, and show in the second simulation study presented later in this article, that the combination of the cluster size-effect size correlation and publication bias operating on *p*-values reproduces results similar to the observed discrepancies in the example (i.e., considerably lower 4PSM and single-level RoBMA estimates, highly variable regression-based approaches). In fact, the simulation study demonstrates that the remaining publication bias-adjustment methods are generally unable to obtain the correct results in those settings.

## Simulation studies

To systematically evaluate the performance of our multilevel RoBMA approach under varying conditions, we perform two simulation studies. First, we adapted the recent comprehensive simulation study conducted by Chen and Pustejovsky (2025) for evaluating multilevel publication bias-adjusted meta-analysis methods. Second, we created a more narrow simulation study inspired by features of the second example to evaluate multilevel publication bias-adjusted meta-analysis and meta-regression methods. In both simulation studies, we included the standard (single-level) publication bias unadjusted Bayesian model-averaged meta-analysis (BMA; Gronau, Heck, Berkhout, Haaf, & Wagenmakers, 2021; Bartoš et al., 2021), robust Bayesian meta-analysis (RoBMA; Bartoš et al., 2022b), and the (here) newly developed three-level counterparts: the publication bias unadjusted multilevel Bayesian model-averaged meta-analysis (BMA (3lvl)), and multilevel robust Bayesian meta-analysis (RoBMA (3lvl)). BMA and BMA (3lvl) meta-analytic ensembles model-average across models with different assumptions about the presence vs. absence of the effect and heterogeneity (e.g., four different sub-models as described in Gronau et al., 2021; Bartoš et al., 2021). RoBMA and RoBMA (3lvl) extend those ensembles with different assumptions about the presence vs. absence of publication bias (e.g., totaling 36 different sub-models as described in Bartoš et al. (2022b); also see end of “Robust Bayesian meta-analysis” section for a complete list of publication bias adjustment components).

We compared the Bayesian methods against several publication bias adjustment methods previously explored by Chen and Pustejovsky (2025). We included two selection models which cannot accommodate a three-level structure due to the computational intractability of implementing three-level modeling within selection models: the three-parameter selection model (3PSM; Hedges, 1992) and four-parameter selection model (4PSM). We further included several multivariate regression-based approaches recently extended to three-level meta-analysis by Chen and Pustejovsky (2025). Specifically, we included the multivariate variants of the PET-PEESE (PET-PEESE-MV; Stanley & Doucouliagos, 2014), and the endogenous kink method (EK-MV; Bom & Rachinger, 2019). These multivariate formulations extend the fixed-effect inverse variance sampling weights to an inverse sampling covariance weights (ICSW) matrix that incorporates within-study correlation, and more accurately reflect the simulation scenarios compared to their univariate counterparts. As frequentist benchmarks, we also included the standard (unadjusted) random-effects meta-analysis (RE) and the within-study dependency adjusted random-effects meta-analysis employing correlated hierarchical effects with inverse sampling covariance weights (RE-CHE-ISCW). For a detailed description of the comparison methods, see Chen and Pustejovsky (2025). The tests of publication bias for the frequentist methods correspond to the test of the regression coefficient of the standard error predictor term from the PET model (i.e., Egger’s test) and the likelihood-ratio test for selection models. For the second part of the second simulation study, we extended the existing code to meta-regression settings.

For brevity, we omit methods identified as less effective in previous simulation studies (e.g., Hong & Reed, 2020; Carter, Schönbrodt, Gervais, & Hilgard, 2019; McShane, Böckenholt, & Hansen, 2016; Bartoš et al., 2022b), such as p-uniform and p-uniform* (van Aert & van Assen, 2021), trim-and-fill (Duval & Tweedie, 2000), PET and PEESE (superseded by PET-PEESE), and fixed-effects meta-analysis, though interested readers can easily generate the comparisons from our openly shared materials.

Both simulations were performed using a distributed Linux cluster^4^ with R (Version 4.1.3, R Core Team, 2021) and the following R packages: the RoBMA package (development version, Bartoš & Maier, 2020) using JAGS (Plummer, 2003) with runjags (Version 2.2.1-7, Denwood, 2016). Note that we used a product space reparameterization of the Bayesian model-averaging (e.g., Lodewyckx et al., 2011), which allows us to compute the Bayesian meta-analytic models considerably faster and without the need for bridge sampling (Meng & Wong, 1996).

### Simulation study based on Chen et al. (2024)

#### Design

The first simulation study follows Chen and Pustejovsky (2025); we generated dependent standardized mean differences based on a two-group experimental design featuring correlated outcome measures. The data-generation process adopted the correlated and hierarchical effects (CHE) working model (Pustejovsky & Tipton, 2022). The simulated publication process depended on the results of a two-sample *t* test that incorporated both publication bias at the study level and selective outcome reporting at the estimate level. See Chen and Pustejovsky (2025) for a detailed explanation of the simulation study and https://osf.io/cxwvm/ for their openly shared code and data.

Due to substantial computational demands associated with MCMC sampling (with a single multilevel RoBMA analysis typically taking between 5 and 15 min), we restricted our simulation study to the following key conditions:

1. Mean effect: $$\mu =$$ (0, 0.2, 0.5)
2. Heterogeneity: $$\tau $$= (0, 0.2, 0.4)
3. Number of estimates per meta-analysis: K = (10, 30, 60, 100)
4. Correlation between nested effect sizes: $\rho$ = (0.2, 0.4, 0.8)
5. Three different publication conditions:
  - No publication bias
  - Publication bias operating on the threshold of statistically significant results in the correct direction (i.e., one-sided $p = 0.05$) with relative publication probabilities of nonsignificant studies relative to significant studies of either 0.80, 0.50, 0.25, or 0.05
  - Publication bias operating on the threshold of statistically significant results in the correct direction and the estimate descriptively pointing in the correct direction (i.e., one-sided $p = 0.05$ and $p = 0.50$) with the following sets of relative publication probabilities: [1.00, 0.50], [0.80, 0.40], [0.50, 0.25], [0.25, 0.125], [0.125, 0.0625], or [0.05, 0.025].

The omitted simulation conditions (included in Chen and Pustejovsky, 2025 but not in this manuscript) were the largest mean effect $\mu$ = 0.8, and small heterogeneity $\tau$ = 0.1. Consequently, our design resulted in $3 \times 3 \times 4 \times 3 \times (1 + 5 + 5) = 1188$ conditions (instead of the original 2304 conditions). To ensure computational feasibility, we evaluated the Bayesian meta-analytic approaches using 300 simulated data sets for each condition (and reused the already-computed results for the remaining methods on the original sample of 2000 data sets).

We evaluated the simulation using root mean square error (RMSE), bias, credible/confidence interval coverage (coverage), power, and error rate (for formulas see e.g., Table 3 in Siepe et al., 2024). The power and error rate were evaluated using $\alpha = 0.05$ for frequentist and $\text {BF}_{10} = 10$ threshold for Bayesian methods.

#### Results

All reported results are conditional upon successful model convergence, defined as a model providing an effect size estimate, a credible/confidence interval, and a corresponding *p*-value or Bayes factor for the presence of the effect. Among all tested methods, only 3PSM and 4PSM exhibited notable convergence issues, failing in 0.14% and 0.47% of cases, respectively.

**Fig. 4:**
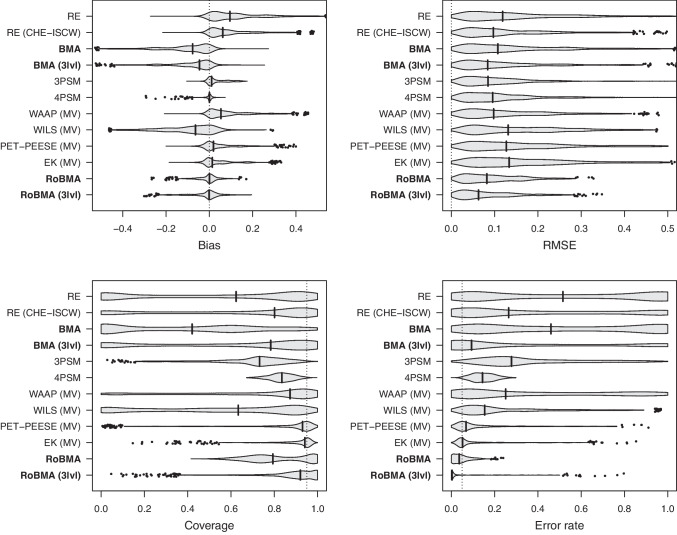
The multilevel version of robust Bayesian meta-analysis, RoBMA (3lvl), performs well in terms of bias, RMSE, coverage, and error rate across the simulation settings. *Note.* *Violin plots* visualize the distribution within the range of 3 standard deviations from the mean; the *vertical line* corresponds to the median; estimates outside the range are visualized as *points*. RE $=$ random effects meta-analysis, RE (CHE-ISCW) $=$ random effects meta-analysis with correlated hierarchical effects and inverse sampling covariance weights, BMA $=$ Bayesian model-averaged meta-analysis (Gronau et al., 2021), BMA (3lvl) $=$ multilevel extension of Bayesian model-averaged meta-analysis, 3PSM = three parameter selection model (Iyengar & Greenhouse, 1988), 4PSM = four parameter selection model (Vevea & Hedges, 1995), PET-PEESE $=$ Precision Effect Test & Precision Effect Estimate with Standard Errors (Stanley & Doucouliagos, 2014), EK $=$ Endogenous Kink (Bom & Rachinger, 2019), RoBMA $=$ Robust Bayesian meta-analysis as in Bartoš et al. (2022b), RoBMA (3lvl) $=$ multilevel extension of RoBMA introduced in this manuscript

Figure 4 summarizes the performance of all examined methods with respect to bias, RMSE, coverage, and error rate of the effect size estimate across all simulation scenarios. Two clear patterns emerge: (1) publication bias-adjusted methods consistently outperform methods without publication bias adjustments; (2) methods accounting explicitly for within-study dependencies (CHE-ISCW, MV, 3lvl) outperform those that ignore such dependencies. Among individual methods, multilevel RoBMA generally surpasses all competitors on these metrics–with one notable exception: 4PSM achieves lower bias. This outcome is unsurprising, as the 4PSM model matches the exact data-generating process employed in the simulation. Although multilevel RoBMA includes the 4PSM as one among several candidate models, uncertainty about model selection results in slightly higher bias relative to directly fitting 4PSM.

Examination of conditions where multilevel RoBMA performed the worst (i.e., conditions outside the violin with low coverage, high error rate, or RMSE) reveals some negative bias in the presence of extreme publication bias (relative publication probabilities of [0.125, 0.0625] and [0.05, 0.025] with two-step selection or 0.05 with one-step selection) and very high heterogeneity (2/3 of the conditions with $\tau = 0.40$). The correlation between nested effect sizes did not seem to influence the negative bias. Nevertheless, multilevel RoBMA exhibits superior performance in terms of RMSE, coverage, and error rate, benefiting from explicit within-study adjustments and the regularizing effect of Bayesian model averaging and prior distributions.

**Fig. 5:**
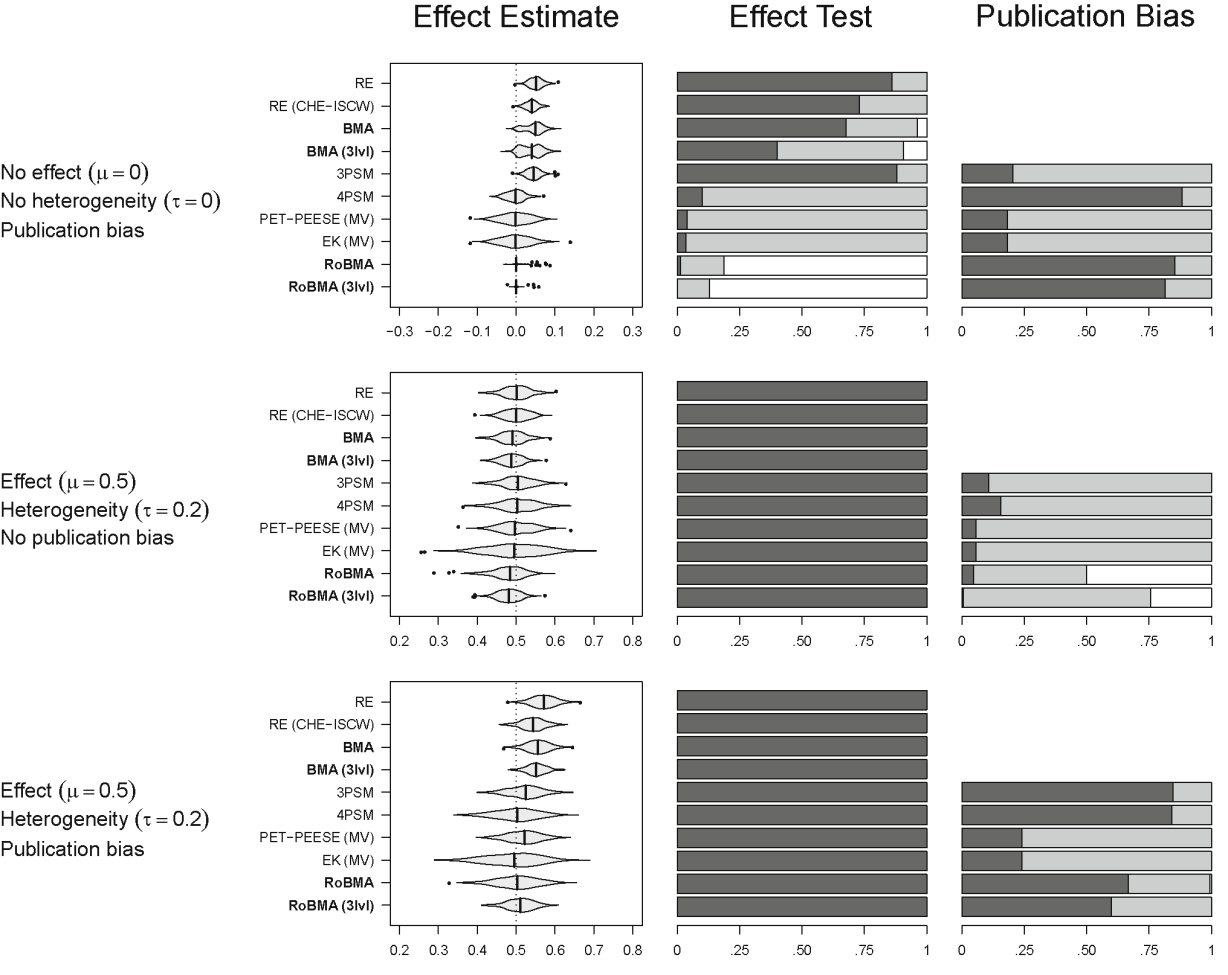
Performance of RoBMA (3lvl) in comparison to other meta-analytic methods for three select cases. *Note*. In the first column, the *vertical line* indicates the true effect size. For the second and third column, the *dark-grey area* indicates evidence for the alternative ($\text {BF}_{10} > 10$ or $p < 0.05$), the *light-grey area* indicates undecided evidence ($10> \text {BF}_{10} > 1/10$ or $p > 0.05$), and the *white area* indicates evidence for the null hypothesis ($\text {BF}_{10} < 1/10$). *Violin plots* visualize the distribution within the range of 3 standard deviations from the mean; the *vertical line* corresponds to the median; estimates outside the range are visualized as *points*. RE $=$ random effects meta-analysis, RE (CHE-ISCW) $=$ random effects meta-analysis with correlated hierarchical effects and inverse sampling covariance weights, BMA $=$ Bayesian model-averaged meta-analysis (Gronau et al., 2021), BMA (3lvl) $=$ multilevel extension of Bayesian model-averaged meta-analysis, 3PSM = three parameter selection model (Iyengar & Greenhouse, 1988), 4PSM = four parameter selection model (Vevea & Hedges, 1995), PET-PEESE $=$ Precision Effect Test & Precision Effect Estimate with Standard Errors (Stanley & Doucouliagos, 2014), EK $=$ Endogenous Kink (Bom & Rachinger, 2019), RoBMA $=$ Robust Bayesian meta-analysis as in Bartoš et al. (2022b), RoBMA (3lvl) $=$ multilevel extension of RoBMA introduced in this manuscript

#### Discussion of selected cases

A comprehensive discussion of all 1188 simulation scenarios exceeds the scope of this manuscript; full results are available at https://osf.io/nd2gv/. Instead, following the approach of Maier et al. (2023), we focus on a selection of common scenarios that facilitate a better understanding of the performance of RoBMA. Figure 5 compares methods across three representative cases:

1. no mean effect ($\mu = 0$), no heterogeneity ($\tau = 0$), and moderate publication bias (selection favoring statistically significant and positive results, relative publication probabilities [0.5, 0.25]),
2. mean effect ($\mu = 0.5$), moderate heterogeneity ($\tau = 0.2$), without publication bias,
3. mean effect ($\mu = 0.5$), moderate heterogeneity ($\tau = 0.2$), with moderate publication bias (selection favoring statistically significant and positive results, relative probabilities [0.5, 0.25]).

All three scenarios involve 100 effect size estimates with a medium within-study correlation ($\rho = 0.4$).

The first row of Fig. 5 illustrates a scenario without mean effect or heterogeneity, but with publication bias (scenario 1). In this scenario, multilevel RoBMA performs similarly to its standard (single-level) counterpart; while the slightly more conservative multilevel version provides a touch more evidence in favor of the absence of the effect, the standard version provides a touch more evidence in favor of publication bias. More notably, both publication-bias-adjusted methods (RoBMA and RoBMA (3lvl)) considerably outperform the unadjusted BMA versions. Bias is substantially reduced, and the adjusted methods typically yield evidence against the presence of an effect. In contrast, the unadjusted BMA approaches often yield inconclusive or even misleading evidence in support of the presence of an effect. The remaining publication bias-adjusted methods, apart from 3PSM, provide unbiased estimates. The 3PSM method also shows a highly inflated error rate for the test of the effect size. Interestingly, from the frequentist methods, 4PSM rejects the null hypothesis of no publication bias in the majority of cases.

The second row of Fig. 5 depicts a scenario with mean effect and heterogeneity, but no publication bias (scenario 2). Employing publication bias adjustments incurs only minimal performance costs for any of the methods; all adjusted and unadjusted models yield highly similar results. All Bayesian approaches exhibit only slight bias attributable mainly to regularization via prior distributions and Bayesian model averaging. Importantly, all methods consistently detect evidence for the true effect. The robust Bayesian meta-analysis models typically detect the absence of publication bias (or yield inconclusive evidence), with only a minute fraction of cases yielding false evidence for publication bias.

The third row of Fig. 5 extends the previous scenario by introducing moderate publication bias (scenario 3). Methods without publication-bias adjustments exhibit upward bias, though they still correctly detect the presence of a true effect. The robust Bayesian meta-analysis approaches (RoBMA and RoBMA (3lvl)) and selection models consistently identify the presence of publication bias in most cases, with only a small proportion of scenarios yielding inconclusive results about the presence of publication bias.

### Simulation study based on Havránková (2025)

#### Design

The second simulation study is inspired by features of the second example (“Relationship Between Beauty and Professional Success”), in particular, the observation that studies which report more effect size estimates tend to have smaller average effect sizes, i.e., the cluster size-effect size correlation. The goal of the simulation study was to validate the meta-regression within the three-level RoBMA. Further, we aimed to assess how the cluster size-effect size correlation documented in Example 2 impacts the performance of meta-analytic and meta-regression methods. Such correlations may be common in practice, for instance, as reviewers and readers may demand more evidence to corroborate a small effect size; it is likely that studies with smaller effects would include more replications of each effect within each primary study. As such, we varied the following parameters,

1. Effect size
  - Meta-analysis: mean effect $\mu = (0, 3)$
  - Meta-regression: moderator-level effects $\mu = ([3, 3, 3], [0, 3, 4])$
2. Cluster size-effect size correlation: $\psi = (-0.25, 0.00)$
3. Two different publication conditions:
  - No publication bias/selective outcome reporting
  - Selective outcome reporting operating on the threshold of statistically significant results in the correct direction, marginally significant results in the correct direction, and the estimate descriptively pointing in the correct direction (i.e., one-sided $p = 0.025$, $p = 0.05$ and $p = 0.50$) with the following relative publication bias probabilities: [1.00, 0.90, 0.50, 0.20]

while keeping the remaining features fixed at either descriptive statistics of the data (i.e., number of studies: 50, marginal distribution of number of estimates per study: log $N \sim \text {Normal}(2.50, 0.95)$, distribution of standard errors of the estimates: log se $\sim \text {Normal}(0.80, 1.05)$) or slightly modified estimates from the multilevel RoBMA meta-regression model (total heterogeneity: $\tau = 4.5$, variance allocation: $\rho = 0.8$).

The data were simulated as follows: first, the joint distribution of study-level effects and the log number of estimates per study was drawn from a multivariate normal distribution. Second, the log standard errors of the estimates were generated from a normal distribution. Third, the observed effect size estimates were generated from a) a normal distribution with the given study-level effect and sampling variance based on the within-study heterogeneity and standard errors in the case of no selective outcome reporting, or b) a weighted normal distribution with the given study-level effect, sampling variance based on the within-study heterogeneity and standard errors, significance thresholds, and relative publication probabilities. As such, the selective outcome reporting condition corresponds to publication bias at the estimate level. Comparison of the effect sizes and test-statistics distribution in the example and under the simulation condition matching the example ($\mu = 3$, $\beta = [-3, 1, 2]$, $\psi = -0.25$, and the presence of selective outcome reporting) verified a close similarity of the data generating model and the observed data (see Fig. 10 in the Appendix).

We directly display the distribution of the pooled effect size estimates (for meta-analysis), estimated marginal means (for meta-regression), and hypothesis tests as in the selected cases of the first simulation study. The power and error rate were evaluated using $\alpha = 0.05$ for frequentist and $\text {BF}_{10} = 10$ threshold for Bayesian methods. We apply the same prior rescaling for Bayesian methods as in the second example.

#### Meta-analysis results

**Fig. 6:**
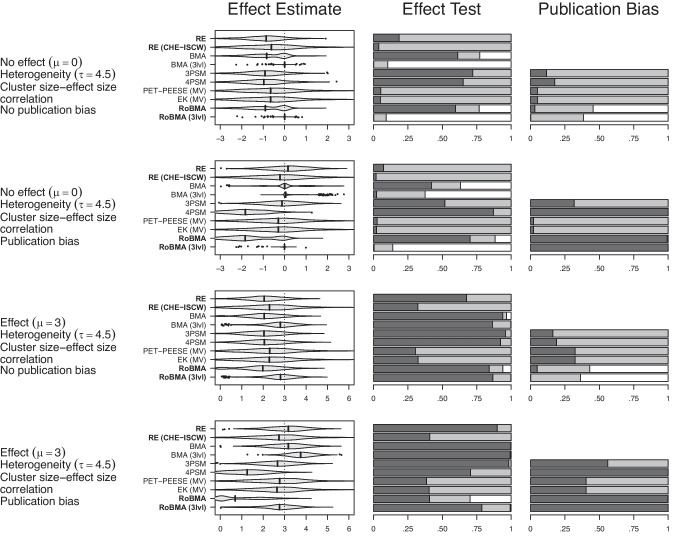
Performance of RoBMA (3lvl) in comparison to other meta-analytic methods in simulation study based on Havránková et al. (2025). *Note*. In the first column, the *vertical line* indicates the true effect size. For the second and third column, the *dark-grey area* indicates evidence for the alternative ($\text {BF}_{10} > 10$ or $p < 0.05$), the *light-grey area* indicates undecided evidence ($10> \text {BF}_{10} > 1/10$ or $p > 0.05$), and the *white area* indicates evidence for the null hypothesis ($\text {BF}_{10} < 1/10$). *Violin plots* visualize the distribution within the range of 3 standard deviations from the mean; the *vertical line* corresponds to the median; estimates outside the range are visualized as *points*. RE $=$ random effects meta-analysis, RE (CHE-ISCW) $=$ random effects meta-analysis with correlated hierarchical effects and inverse sampling covariance weights, BMA $=$ Bayesian model-averaged meta-analysis (Gronau et al., 2021), BMA (3lvl) $=$ multilevel extension of Bayesian model-averaged meta-analysis, 3PSM = three parameter selection model (Iyengar & Greenhouse, 1988), 4PSM = four parameter selection model (Vevea & Hedges, 1995), PET-PEESE $=$ Precision Effect Test & Precision Effect Estimate with Standard Errors (Stanley & Doucouliagos, 2014), EK $=$ Endogenous Kink (Bom & Rachinger, 2019), RoBMA $=$ Robust Bayesian meta-analysis as in Bartoš et al. (2022b), RoBMA (3lvl) $=$ multilevel extension of RoBMA introduced in this manuscript

Figure 6 displays results from the meta-analytic settings under the presence of a negative cluster size-effect size correlation (see Fig. 8 in the Appendix for corresponding results under no cluster size-effect size correlation).

**Fig. 7:**
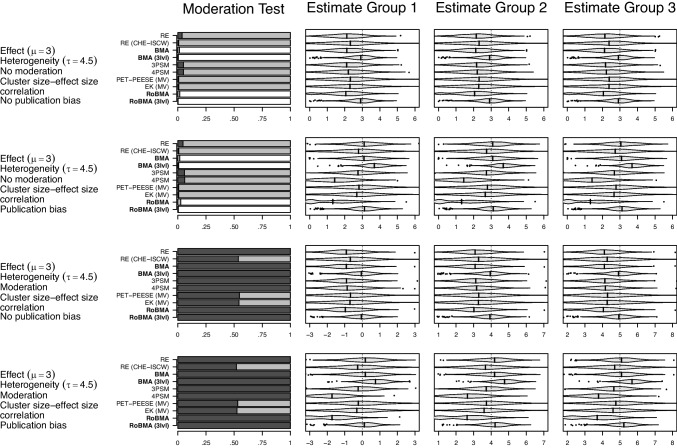
Performance of RoBMA meta-regression (3lvl) in comparison to other meta-regression methods in simulation study based on Havránková et al. (2025). *Note*. In the first column, the *dark-grey area* indicates evidence for the alternative ($\text {BF}_{10} > 10$ or $p < 0.05$), the *light-grey area* indicates undecided evidence ($10> \text {BF}_{10} > 1/10$ or $p > 0.05$), and the *white area* indicates evidence for the null hypothesis ($\text {BF}_{10} < 1/10$). In the remaining columns, the *vertical line* indicates the true effect size. *Violin plots* visualize the distribution within the range of 3 standard deviations from the mean; the *vertical line* corresponds to the median; estimates outside the range are visualized as points. RE $=$ random effects meta-regression, RE (CHE-ISCW) $=$ random effects meta-regression with correlated hierarchical effects and inverse sampling covariance weights, BMA $=$ Bayesian model-averaged meta-regression (Gronau et al., 2021), BMA (3lvl) $=$ multilevel extension of Bayesian model-averaged meta-regression, 3PSM = three parameter selection meta-regression (Iyengar & Greenhouse, 1988), 4PSM = four parameter selection meta-regression (Vevea & Hedges, 1995), PET-PEESE $=$ Precision Effect Test & Precision Effect Estimate with Standard Errors meta-regression (Stanley & Doucouliagos, 2014), EK $=$ Endogenous Kink meta-regression (Bom & Rachinger, 2019), RoBMA $=$ Robust Bayesian meta-regression as in Bartoš et al. (2022b), RoBMA (3lvl) $=$ multilevel extension of RoBMA introduced in this manuscript

**Fig. 8:**
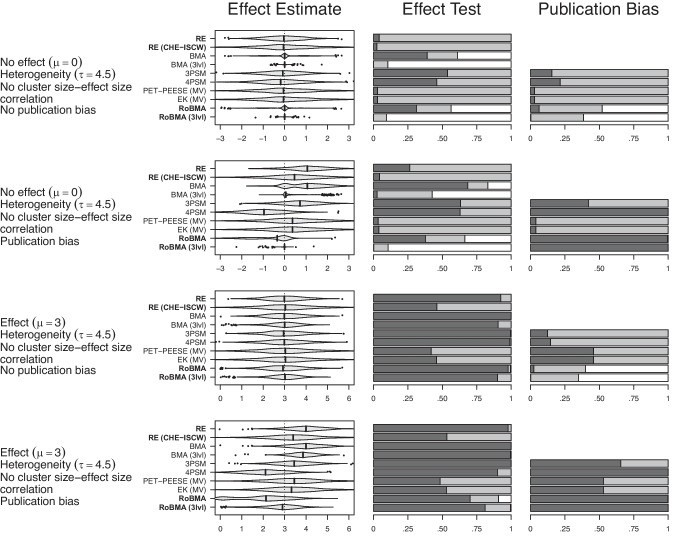
Performance of RoBMA (3lvl) in comparison to other meta-analytic methods in simulation study based on Havránková et al. (2025). *Note*. In the first column, the *vertical line* indicates the true effect size. For the second and third column, the *dark-grey area* indicates evidence for the alternative ($\text {BF}_{10} > 10$ or $p < 0.05$), the *light-grey area* indicates undecided evidence ($10> \text {BF}_{10} > 1/10$ or $p > 0.05$), and the *white area* indicates evidence for the null hypothesis ($\text {BF}_{10} < 1/10$). *Violin plots* visualize the distribution within the range of 3 standard deviations from the mean; the vertical line corresponds to the median; estimates outside the range are visualized as points. RE $=$ random effects meta-analysis, RE (CHE-ISCW) $=$ random effects meta-analysis with correlated hierarchical effects and inverse sampling covariance weights, BMA $=$ Bayesian model-averaged meta-analysis (Gronau et al., 2021), BMA (3lvl) $=$ multilevel extension of Bayesian model-averaged meta-analysis, 3PSM = three parameter selection model (Iyengar & Greenhouse, 1988), 4PSM = four parameter selection model (Vevea & Hedges, 1995), PET-PEESE $=$ Precision Effect Test & Precision Effect Estimate with Standard Errors (Stanley & Doucouliagos, 2014), EK $=$ Endogenous Kink (Bom & Rachinger, 2019), RoBMA $=$ Robust Bayesian meta-analysis as in Bartoš et al. (2022b), RoBMA (3lvl) $=$ multilevel extension of RoBMA introduced in this manuscript

Examining the effect size estimates (first column) reveals a surprising pattern: all methods except for the multilevel version of BMA and RoBMA show substantial negative bias if selective outcome reporting is absent (first and third rows). If selective outcome reporting is present (second and fourth rows), multilevel BMA begins to overestimate the effect size, while multilevel RoBMA properly adjusts the estimate. The remaining methods exhibit a mixed pattern of results in the presence of selective outcome reporting due to the negative bias under the negative cluster size-effect size correlation and positive bias under selective outcome reporting (see the second and fourth rows of Fig. 8 in the Appendix for no cluster size-effect size correlation). As such, only the multilevel RoBMA seems to be able to recover the true estimate under all conditions.

The second column reflects the consequences of these biases for the test of effect size. In the simulated settings, only multilevel RoBMA is able to consistently obtain evidence for (first and second rows) and against (third and fourth rows) the effect, regardless of selective outcome reporting or the cluster size-effect size correlation. The single-level RoBMA often provides incorrect evidence for the presence of an effect in its absence and incorrect evidence for the absence of the effect in its presence. Additionally, selection models (3PSM and 4PSM) have greatly inflated the type I error rate, regardless of the condition, while multivariate PET-PEESE and EK lack the power to detect the true effect.

The third column shows that multilevel RoBMA is able to consistently find evidence for either the absence (first and third rows) or the presence (second and fourth rows) of publication bias, and single-level RoBMA performs similarly across the conditions. Among the remaining methods, only 4PSM detects publication bias in the majority of cases, whereas all others fail to indicate its presence.

#### Meta-regression results

**Fig. 9:**
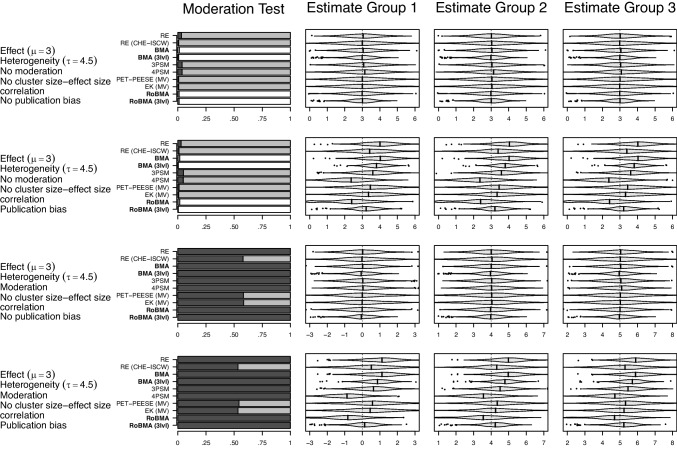
Performance of RoBMA meta-regression (3lvl) in comparison to other meta-regression methods in simulation study based on Havránková et al. (2025). *Note*. In the first column, the *dark-grey area* indicates evidence for the alternative ($\text {BF}_{10} > 10$ or $p < 0.05$), the *light-grey area* indicates undecided evidence ($10> \text {BF}_{10} > 1/10$ or $p > 0.05$), and the *white area* indicates evidence for the null hypothesis ($\text {BF}_{10} < 1/10$). *Violin plots* visualize the distribution within the range of 3 standard deviations from the mean; the *vertical line* corresponds to the median; estimates outside the range are visualized as points. In the remaining columns, the *vertical line* indicates the true effect size. RE $=$ random effects meta-regression, RE (CHE-ISCW) $=$ random effects meta-regression with correlated hierarchical effects and inverse sampling covariance weights, BMA $=$ Bayesian model-averaged meta-regression (Gronau et al., 2021), BMA (3lvl) $=$ multilevel extension of Bayesian model-averaged meta-regression, 3PSM = three parameter selection meta-regression (Iyengar & Greenhouse, 1988), 4PSM = four-parameter selection meta-regression (Vevea & Hedges, 1995), PET-PEESE $=$ Precision Effect Test & Precision Effect Estimate with Standard Errors meta-regression (Stanley & Doucouliagos, 2014), EK $=$ Endogenous Kink meta-regression (Bom & Rachinger, 2019), RoBMA $=$ Robust Bayesian meta-regression as in Bartoš et al. (2022b), RoBMA (3lvl) $=$ multilevel extension of RoBMA introduced in this manuscript

**Fig. 10:**
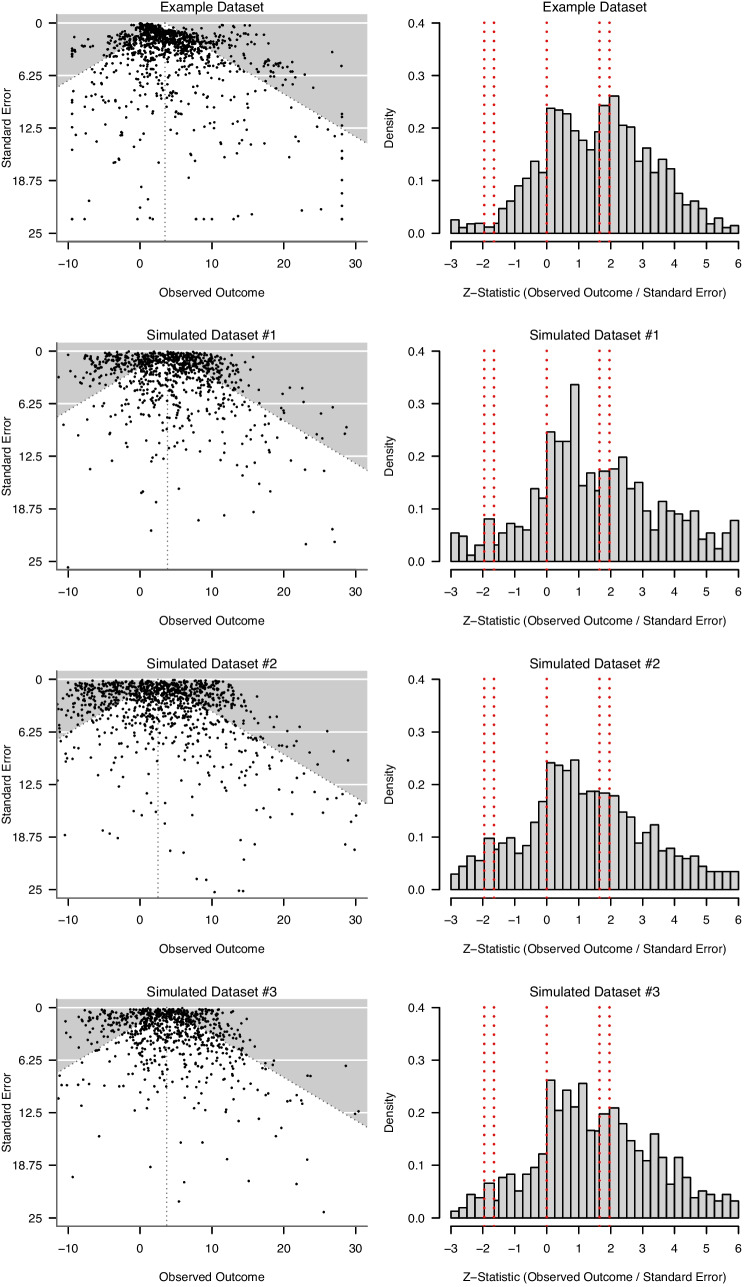
Comparison of the example dataset and simulated datasets from simulation study based on Havránková et al. (2025)

Figure 7 displays results from the meta-regression settings under the presence of a negative cluster size-effect size correlation (see Fig. 9 in the Appendix for corresponding results under no cluster size-effect size correlation).

Examining the moderation test (first column) reveals that all methods, except for the random effects meta-regression with correlated hierarchical effects (RE CHE-ISCW) and the multivariate PET-PEESE and EK meta-regressions, always detect the moderation when it is present, regardless of the condition. In addition, BMA and RoBMA methods also provide compelling evidence against the presence of moderation when it is absent (first and second rows).

The remaining columns (second, third, and fourth) indicate the same pattern of bias in the estimated marginal means as we observed for the effect size estimate in the meta-analysis simulation. All methods, apart from RoBMA, show substantial bias due to at least one of the factors; the negative cluster size-effect size correlation leads to negative bias for all methods except multilevel BMA, and RoBMA, while selective outcome reporting leads to positive bias for all methods except RoBMA. The combination of the two factors results in a mix of biases that sometimes cancel out (which is why the negative bias is often reduced under publication bias: rows 2 and 4).

## Conclusion

In this manuscript, we extended robust Bayesian meta-analysis and meta-regression (RoBMA) to explicitly accommodate three-level hierarchical structures. This extension allows researchers to benefit from Bayesian model averaging and principled publication bias adjustments in settings with multiple estimates originating from the same study, a scenario that characterizes the majority of real-world meta-analyses (Wu et al., 2025). Consequently, the extension to three-level structures substantially broadens the applicability and practical utility of RoBMA.

We demonstrated how to apply the methodology using the RoBMA R package and JASP in two empirical examples and evaluated its performance in two simulation studies. Both simulations clearly highlight that the newly proposed multilevel version of RoBMA performs better than the original method if within-study dependencies are present (also see Bartoš, Pawel, & Siepe, 2025b, for a neutral performance comparison of the included methods). Furthermore, the second simulation study uncovered severe issues with the remaining methods in the presence of cluster size-effect size correlation alone. Importantly, neither of the simulation studies should be viewed as conclusive evidence for good method performance for all possible applications to real datasets. The first simulation, adapted from Chen and Pustejovsky (2025), based the data-generating model (incorporating both publication bias and selective outcome reporting) on a four-parameter selection model and inadvertently benefited from selection models. The second simulation, modeled after the example dataset, also based the data-generating model (incorporating only selective outcome reporting) on a selection model included in RoBMA and inadvertently benefited our method.

Future methodological developments should consider a more extensive examination of the different effects of publication bias operating on the study level and selective outcome reporting on the estimate level (see e.g., Chen & Pustejovsky, 2025), impact of more informed prior distributions, and a more comprehensive evaluation of tests for detecting publication bias (Fernández-Castilla et al., 2021; Park et al., 2025; Rodgers & Pustejovsky, 2021). The Bayesian methodology itself could be further developed to incorporate more complex hierarchical structures, such as four-level meta-analyses, multiple random-effects components, autoregressive dependencies, or mixtures of latent classes. Although the conceptual framework outlined here remains directly applicable, additional software advances will be required to attain the complexity and flexibility currently available in frequentist meta-analytic packages (e.g., metafor; Viechtbauer, 2010).

An even more challenging problem involves extending Bayesian meta-analytic models to handle within-study dependencies arising from multiple outcome measures or shared control groups. Currently, the most straightforward, though admittedly suboptimal, solution involves pooling effect sizes and standard errors according to established rules (e.g., Borenstein et al., 2009). Future research efforts could focus on developing multivariate generalizations of our multilevel RoBMA approach, thereby providing a more principled and robust solution.

We anticipate that the multilevel extension of RoBMA will facilitate broader adoption of Bayesian approaches in meta-analysis, and we invite further methodological advancements aimed at developing principled, flexible, transparent, and rigorous analytical tools.

## Data Availability

The data and R scripts for performing the analyses and simulation study are openly available on OSF at https://osf.io/nd2gv/.

## Appendix A: Additional example results

**Table 1:** Conflicting conclusions of different publication bias adjustment methods when applied to the secondary benefits of family participation in treatment for childhood disorders example data

Method	Effect	Publication bias
BMA	$0.22 \,[ 0.19, 0.26]$	—
	$$\text {BF}_{10} > 10^6$$	
BMA (3lvl)	$0.25 \,[ 0.20, 0.30]$	—
	$$\text {BF}_{10} > 10^6$$	
RoBMA	$0.00 \,[ 0.00, 0.00]$	$\text {BF}_{\text {bias}} > 10^6$
	$$\text {BF}_{10} = 0.04$$	
RoBMA (3lvl)	$0.05 \,[ 0.00, 0.17]$	$\text {BF}_{\text {bias}} > 10^6$
	$$\text {BF}_{10} = 0.93$$	
RE	$0.22 \,[ 0.17, 0.27]$	—
	$$p < 0.001$$	
RE (CHE-ISCW)	$0.17 \,[ 0.11, 0.22]$	—
	$$p < 0.001$$	
3PSM	$0.37 \,[ 0.30, 0.45]$	$p < 0.001$
	$$p < 0.001$$	
4PSM	$0.18 \,[ 0.07, 0.30]$	$p < 0.001$
	$$p = 0.002$$	
PET-PEESE (MV)	$-0.04 \,[-0.29, 0.20]$	$p = 0.041$
	$$p = 0.698$$	
EK (MV)	$-0.04 \,[-0.29, 0.20]$	$p = 0.041$
	$$p = 0.698$$	

*Note.* RE $=$ random effects meta-analysis, RE (CHE-ISCW) $=$ random effects meta-analysis with correlated hierarchical effects and inverse sampling covariance weights, BMA $=$ Bayesian model-averaged meta-analysis (Gronau et al., 2021), BMA (3lvl) $=$ multilevel extension of Bayesian model-averaged meta-analysis, 3PSM = three-parameter selection model (Iyengar & Greenhouse, 1988), 4PSM = four parameter selection model (Vevea & Hedges, 1995), PET-PEESE $=$ Precision Effect Test & Precision Effect Estimate with Standard Errors (Stanley & Doucouliagos, 2014), EK $=$ Endogenous Kink (Bom & Rachinger, 2019), RoBMA $=$ Robust Bayesian meta-analysis as in Bartoš et al. (2022b), RoBMA (3lvl) $=$ multilevel extension of RoBMA introduced in this manuscript. PET-PEESE (MV) and EK (MV) results coincide in the example

**Table 2:** Conflicting conclusions of different publication bias adjustment methods when applied to the relationship between beauty and professional success example data

Method	Average effect	Publication bias	Moderation	No contact	Some contact	Direct contact
BMA	$3.4 \,[3.1, 3.7]$	—	$\text {BF}_{\text {mod}} = 0.76$	$3.1 \,[ 2.2, 3.7]$	$3.4 \,[ 3.0, 3.7]$	$3.7 \,[3.2, 4.3]$
	$\text {BF}_{10} > 10^6$			$\text {BF}_{10} > 10^6$	$\text {BF}_{10} > 10^6$	$\text {BF}_{10} > 10^6$
BMA (3lvl)	$4.1 \,[3.1, 5.2]$	—	$\text {BF}_{\text {mod}} > 10^6$	$2.1 \,[ 0.7, 3.4]$	$5.0 \,[ 3.5, 6.4]$	$5.4 \,[4.2, 6.6]$
	$\text {BF}_{10} > 10^6$			$\text {BF}_{10} = 4.49$	$\text {BF}_{10} > 10^6$	$\text {BF}_{10} > 10^6$
RoBMA	$0.0 \,[0.0, 0.7]$	$\text {BF}_{\text {bias}} > 10^6$	$\text {BF}_{\text {mod}} = 19999$	$-1.1 \,[ -1.7, -0.3]$	$0.2 \,[ -0.3, 0.9]$	$1.1 \,[0.6, 1.8]$
	$\text {BF}_{10} = 0.07$			$\text {BF}_{10} = 0.79$	$\text {BF}_{10} = 0.02$	$\text {BF}_{10} > 10^6$
RoBMA (3lvl)	$3.0 \,[1.9, 4.1]$	$\text {BF}_{\text {bias}} > 10^6$	$\text {BF}_{\text {mod}} = 10^6$	$0.8 \,[ -0.6, 2.2]$	$4.0 \,[ 2.5, 5.6]$	$4.2 \,[3.0, 5.6]$
	$\text {BF}_{10} > 10^6$			$\text {BF}_{10} = 0.10$	$\text {BF}_{10} > 10^6$	$\text {BF}_{10} > 10^6$
RE	$3.3 \,[ 2.4, 4.3]$	—	$p = 0.6047$	$2.7 \,[ 0.4, 5.0]$	$3.3 \,[ 2.4, 4.3]$	$3.9 \,[ 2.6, 5.3]$
	$p < 0.001$			$p = 0.020$	$p < 0.001$	$p < 0.001$
RE (CHE-ISCW)	$0.5 \,[-0.2, 1.2]$	—	$p = 0.7706$	$0.1 \,[-0.7, 0.8]$	$1.1 \,[-0.8, 3.0]$	$0.3 \,[-0.3, 0.9]$
	$p = 0.154$			$p = 0.814$	$p = 0.247$	$p = 0.262$
3PSM	$2.6 \,[ 2.2, 3.0]$	$p < 0.001$	$p < 0.001$	$1.9 \,[ 1.3, 2.6]$	$2.6 \,[ 2.1, 3.1]$	$3.2 \,[ 2.8, 3.7]$
	$p < 0.001$			$p < 0.0001$	$p < 0.001$	$p < 0.001$
4PSM	$0.9 \,[ 0.3, 1.5]$	$p < 0.001$	$p < 0.001$	$-0.1 \,[-1.0, 0.8]$	$1.0 \,[ 0.2, 1.7]$	$1.9 \,[ 1.1, 2.6]$
	$p = 0.005$			$p = 0.772$	$p = 0.009$	$p < 0.001$
PET-PEESE (MV)	$0.5 \,[-0.2, 1.1]$	$p < 0.001$	$p = 0.706$	$0.1 \,[-0.3, 0.5]$	$1.1 \,[-0.7, 2.8]$	$0.3 \,[-0.3, 0.9]$
	$p = 0.128$			$p = 0.681$	$p = 0.225$	$p = 0.286$
EK (MV)	$0.5 \,[-0.2, 1.1]$	$p < 0.001$	$p = 0.706$	$0.1 \,[-0.3, 0.5]$	$1.1 \,[-0.7, 2.8]$	$0.3 \,[-0.3, 0.9]$
	$p = 0.128$			$p = 0.681$	$p = 0.225$	$p = 0.286$

*Note.* RE $=$ random effects meta-analysis, RE (CHE-ISCW) $=$ random effects meta-analysis with correlated hierarchical effects and inverse sampling covariance weights, BMA $=$ Bayesian model-averaged meta-analysis (Gronau et al., 2021), BMA (3lvl) $=$ multilevel extension of Bayesian model-averaged meta-analysis, 3PSM = three-parameter selection model (Iyengar & Greenhouse, 1988), 4PSM = four-parameter selection model (Vevea & Hedges, 1995), PET-PEESE $=$ Precision Effect Test & Precision Effect Estimate with Standard Errors (Stanley & Doucouliagos, 2014), EK $=$ Endogenous Kink (Bom & Rachinger, 2019), RoBMA $=$ Robust Bayesian meta-analysis as in Bartoš et al. (2022b), RoBMA (3lvl) $=$ multilevel extension of RoBMA introduced in this manuscript. PET-PEESE (MV) and EK (MV) results coincide in the example

## Footnotes

1 The term “three-level” indicates that an additional hierarchical layer is added to the standard two-level random-effects model to account explicitly for within-study dependency.

2 An alternative approach involves cluster-robust standard errors (Hedges, Tipton, & Johnson, 2010); however, we do not pursue this option since no Bayesian counterpart currently exists.

3 Our attempts to implement this fully marginalized version in JAGS and FORTRAN revealed severe computational limitations; reliable estimation was only feasible with up to three effect sizes per study. This computational burden arises because the normalizing constant involves $(w+1)^{J_i}$ multivariate normal integrals, where *w* denotes the number of steps in the weight function. Such computational challenges may explain why, to the best of our knowledge, multilevel selection models have not been applied in practice.

4 The first simulation took approximately 1500 CPU hours, the second simulation took approximately 250 CPU hours.

## Code Availability

The software is implemented in the RoBMA R package available at https://CRAN.R-project.org/package=RoBMA and JASP Meta-Analysis module available at http://github.com/jasp-stats/jaspMetaAnalysis/.

## Declarations

### Author Note

All code and data underlying this manuscript are available at https://osf.io/nd2gv/.
